# The emerging roles of piezo1 channels in animal models of multiple sclerosis

**DOI:** 10.3389/fimmu.2022.976522

**Published:** 2022-09-13

**Authors:** Kai Yang, Xueai He, Zhengqi Wu, Yimeng Yin, Hanyu Pan, Xinyue Zhao, Taolei Sun

**Affiliations:** ^1^ School of Chemistry, Chemical Engineering and Life Science, Wuhan University of Technology, Wuhan, China; ^2^ State Key Laboratory of Advanced Technology for Materials Synthesis and Processing, Wuhan University of Technology, Wuhan, China

**Keywords:** piezo channels, mechanotransduction, membrane dome mechanism, membrane footprint hypothesis, multiple sclerosis

## Abstract

Multiple sclerosis (MS) is a chronic inflammatory, demyelinating, and neurodegenerative disease in the central nervous system (CNS). Its pathogenesis is quite complex: Accumulated evidence suggests that biochemical signals as well as mechanical stimuli play important roles in MS. In both patients and animal models of MS, brain viscoelasticity is reduced during disease progression. Piezo mechanosensitive channels are recently discovered, and their three-dimensional structure has been solved. Both the membrane dome mechanism and the membrane footprint hypothesis have been proposed to explain their mechanosensitivity. While membrane-mediated forces alone appear to be sufficient to induce Piezo gating, tethers attached to the membrane or to the channel itself also seem to play a role. Current research indicates that Piezo1 channels play a key role in multiple aspects of MS pathogenesis. Activation of Piezo1 channels in axon negatively regulates CNS myelination. in addition, the inhibition of Piezo1 in CD4+ T cells and/or T regulatory cells (Treg) attenuates experimental autoimmune encephalitis (EAE) symptoms. Although more work has to be done to clarify the roles of Piezo1 channels in MS, they might be a promising future drug target for MS treatment.

## The introduction of multiple sclerosis

Multiple sclerosis (MS) is a chronic inflammatory, demyelinating, and neurodegenerative disease in the central nervous system (CNS) (1). In general, the disease begins when patients are in their third decade of life with a relapsing and remitting clinical course (RRMS). It then slowly converts to a serious and irreversible neurological impairment, which is called secondary-progressive MS (SPMS) (1). Other patients show rapidly progressive neurological deficits, which belong to the primary-progressive form of MS (PPMS) (2). MS is heterogeneous and influenced by both genetic and environmental factors. The most well-established environmental risk factors are Epstein–Barr virus (EBV) infection and low vitamin D levels (3, 4). Several studies suggest that low vitamin D levels are linked to increased risk of MS (3). Although the beneficial mechanisms of vitamin D on MS are not fully understood, some studies suggest that the active form of vitamin D modulates immune functions (5). EBV infection has also been suggested to be associated with MS (4), but the mechanism by which EBV infection increases the risk of MS is not understood. It is possible that molecular mimicry produced by EBV infection leads to the generation of cross-reactive T cells, which increases the occurrence of MS. Genome-​wide association studies (GWAS) have identified more than 200 genetic risk variants for MS, all linked to immune functions (6). Although each variant alone only slightly increases a patient’s genetic susceptibility to MS, combinations of these variants significantly affect the risk of MS. Among these risk variants, polymorphisms in human leukocyte antigen (HLA) class I and class II genes contribute to the highest risk of MS (1).

The pathological hallmark of MS is focal plaques in white matter (WM) and grey matter (GM) of the brain and spinal cord. They are caused by the infiltration of immune cells across the dysregulated blood–brain barrier (BBB), which leads to inflammation and demyelination, reactive gliosis, and axonal degeneration. However, the triggering event that initiates this autoimmune response in MS is not understood. Two hypotheses have been proposed to explain the etiopathology of MS (7, 8). One is called the autoimmune (outside-in) hypothesis (9), which claims that MS is caused by the activation of peripheral autoreactive effector CD4 T cells, which migrate to the CNS and initiate the disease process. Animal models of EAE have been created successfully based on this hypothesis (10). In addition, either immunodepleting agents (11) or lymphocyte anti-trafficking agents (12) are widely used to treat MS; these drugs all target peripheral immunological processes and effectively shut down most of the focal inflammatory events. The neurodegenerative (inside-out) hypothesis states that inflammation begins in the CNS (13, 14). The autoreactive effector CD4+ T cells are locally reactivated by antigen-presenting cells (APCs) in the CNS, which recruit additional T cells and macrophages to cause the inflammatory lesion.

## The mechanobiology of the brain in MS

The pathogenesis of MS is quite complex: Accumulated evidence suggests that biochemical signals as well as mechanical stimuli play important roles in the disease. Over the past several decades, research on MS has been dominated by electrophysiological, biochemical, molecular, genetic, and imaging studies. The mechanical forces that influence MS pathogenesis remain largely unexplored. The brain is a viscoelastic material and is one of the softest tissues in the body. Usually, Young’s modulus, the elastic modulus (*E*), or stiffness is used to describe the resistance (or tendency) of brain to deform in response to mechanical force (15).

Measurement of brain stiffness has been especially difficult because it is quite small. Along with the advancement of techniques, mechanical measurements of the brain and spinal cord have been done using magnetic resonance elastography (MRE) and atomic force microscopy (AFM). MRE measures tissue stiffness by imaging their responses to sound (shear) waves propagated through the tissues. This technique is non-invasive and can be used to analyze the mechanical properties of brain *in vivo*. However, it can only measure the mechanical properties of large samples (16). AFM indentation, on the contrary, uses a small radius tip to locally apply compressive stress to the samples and then measures their mechanical responses. Therefore, it is more suitable to measure the mechanical properties of the different small regions of the brain (17).

It is well accepted that the brain exhibits heterogeneous mechanical properties in different areas (18–26). In the rat dentate gyrus, the subgranular zone (SGZ) and hilus (H) have similar stiffness, while the granule cell layer (GCL) is at least twofold stiffer (27). Furthermore, GM consists primarily of neuronal cell bodies, dendrites, and unmyelinated axons, while WM is made up of myelinated axons, oligodendrocytes, astrocytes, and microglia. Although some studies have observed that GM is significantly stiffer than WM (18, 28), others have claimed that they have equal stiffness (29, 30), and yet others have reported that WM is stiffer than GM (19, 20, 23). These differences may arise from the different experimental setups, sample preparation, post-mortem time and testing conditions (18, 23).

In several human studies, researchers have reported that brain viscoelasticity is reduced during normal aging (31), Parkinson’s disease (32), and Alzheimer’s disease (33). Not surprisingly, there is a positive correlation between reduced brain stiffness and the clinical score in patients with MS. For patients with mild relapsing-remitting MS, MRE revealed that there is a significant 13% reduction in parenchymal stiffness compared to healthy controls (34). Furthermore, patients with primary and secondary chronic-progressive MS showed a pronounced reduction in cerebral shear elasticity compared with patients with early relapsing-remitting MS (35). In addition, patients with clinically isolated syndrome (CIS), which indicates the first clinical onset of potential MS, also exhibited decreased brain viscoelasticity (36).

A further confirmation of these clinical results has come from studies in MS animal models. In the myelin proteolipid protein (PLP)–induced EAE animal model using SJL (“Swiss Jim Lambert”) mice, which is characterized by a relapsing-remitting course, viscoelasticity of the brain is reduced during the acute phase of EAE. However, this reduction is reversible: Brain viscoelasticity returns to the baseline level during the recovery phase of EAE (37). Brain viscoelasticity has also been investigated in the adoptive transfer EAE model using SJL mice. It was induced by the injection of pre-activated lymphocytes, which are stimulated by PLP. This EAE model demonstrates more severe symptoms with large cerebral infiltrations. Brain stiffness in this EAE model is significantly reduced in the areas of inflammation in the brain (38).

In another study, researchers produced a chronic EAE model by immunizing both C57BL/6 mice and interferon γ (IFNγ) knockout mice with myelin oligodendrocyte glycoprotein peptide 35–55 (MOG_35–55_). They found no change in the brain viscoelasticity of C57BL/6 mice, which is in contrast to the PLP-induced EAE SJL mouse model (37). However, brain viscoelasticity of the IFNγ knockout mouse is altered during the course of the disease (39). Researchers have also collected spinal cords of chronic EAE mice during pre-onset, onset, peak, and chronic disease phases, and measured their mechanical properties using AFM. They found increased stiffness of WM during the onset phase, maintenance of this increased stiffness during the peak phase, and a decrease in the chronic phase, while GM shows no changes (40). In this study, greater WM rigidity of EAE mice than that of the control mice during the onset and peak phases of the disease is reported, this result is not consistent with the MRE studies, which have shown that brain tissue is softer in patients with MS and EAE mice compared with healthy controls. The reason for this discrepancy requires further investigation.

Brain stiffness is positively correlated with the myelin content (24). In the cuprizone model of MS, it showed that the stiffness in the corpus callosum is increased initially, followed by a decrease at the 12-week time point. The authors proposed that the decrease in brain stiffness is caused by progressive demyelination and global extracellular matrix (ECM) degradation (41). In addition, acute and chronic demyelinated CNS lesions show the opposite changes in stiffness. Acutely demyelinated lesions in both the lysolecithin and cuprizone models, which have the ability to remyelinate spontaneously, display reduced stiffness compared with healthy tissue. On the contrary, tissue from a chronic cuprizone model which failed to remyelinate, is stiffer than healthy control (42).

## The introduction of piezo channels

The hypothesis that ion channels act as mechanical sensors has been suggested for decades, but their identity in mammals remained unknown until 2010. Using RNA interference (RNAi)-mediated knockdown of selected genes in the mouse Neuro2A neuroblastoma cell line, Coste et al. identified a single gene, Piezo1, that is required for mechanically activated cationic currents. Further sequence homology analysis revealed its analog, Piezo2. Piezo1 channels are highly expressed in the brain and non-sensory tissues including the lung, bladder, and skin. By contrast, Piezo2 channels are predominantly located in sensory tissues, such as dorsal root ganglia (DRG) sensory neurons and Merkel cells (43).

### The structure of piezo channels

Piezo1 is one of the largest receptors for mechanical forces that has been described. It consists of three identical subunits that form one central pore and three long blades extending away from the center (44, 45). The central pore of Piezo1 is responsible for ion conductance while the extracellular blades are responsible for its mechanosensitivity. The beam structure positioned at a 30° angle relative to the membrane plane acts as a lever-like apparatus. Coupling between the distal blades and central pore through the beam structure converts mechanical force into a force used for cation conduction.

The membrane dome mechanism has been proposed to explain the mechanosensitivity of Piezo channels. It claims that the extended blades of Piezo channels have the ability to locally deform lipid membranes into a dome-like shape, which provides energy for Piezo gating (46). Under force, lateral membrane tension flattens the Piezo dome, which increases the energy of the membrane channel system and opens Piezo channels However, the membrane dome model of Piezo gating only considers the shape of the membrane inside Piezo’s perimeter and does not include the effect of the surrounding membrane. To overcome this shortcoming, Haselwandter et al. have proposed the “membrane footprint hypothesis,” which suggests that Piezo1 also deforms the surrounding lipid membrane outside the perimeter of the channel into a curved membrane footprint. This membrane footprint amplifies the tension sensitivity of Piezo1 (47).

### The gating of piezo channels

Two models have been proposed to explain the gating of Piezo channels: The “force through lipid” model claims that bilayer tension directly gates the channel, and the “force through filaments” model suggests that mechanical stimuli are transduced through physical tethers to the cytoskeleton or the ECM (48). These two models are not mutually exclusive: Although some mechanosensitive channels are gated by only one mechanism, both models may contribute to activate Piezo channels.

Piezo1 can be activated by membrane tension alone. Membrane blebs free of cytoskeleton can be generated from Piezo1-transfected HEK293 cells subjected to a hypoosmotic solution. Piezo1 currents evoked by mechanical stimulation can be recorded on these patched blebs. Even when blebbed membranes are pretreated with drugs that disrupt microtubule assembly and the actin cytoskeleton, Piezo1 currents still exist (49). Furthermore, when Piezo1 channels are reconstituted in asymmetric bilayers, their activation can be induced by membrane asymmetry, osmotic stress, and solvent injection (50). These studies provide strong evidence that Piezo1 is inherently mechanosensitive, and mechanical force can be transmitted directly from the membrane to the channel.

The roles of the cytoskeleton and associated scaffolding proteins in the activation of Piezo1 by mechanical force have also been examined. Piezo1 currents are inhibited by the disruption of actin in a heterologous expression system (51). In addition, in Neuro2A cells, the scaffolding protein stomatin-like protein 3 (STOML3) sensitizes Piezo1 channels *via* cholesterol binding and modulation of plasma membrane (PM) stiffness (52, 53).

In conclusion, force from both membrane tension and physical tethers can modulate Piezo1 activity, but which mechanism is dominant depends on the cell type and/or mechanical stimulus (54). Although membrane-mediated forces alone have ability to open Piezo1 channels, tethers attached to the membrane or to the channel itself also play a role in the gating of Piezo1 channels.

## The role of piezo channels in animal models of MS

### Demyelination and axon degeneration

Both demyelination and axon degeneration are pathological hallmarks of MS, and Demyelination occurs in both GM and WM. As myelin is responsible for fast axonal conduction, its loss usually disrupts the communication between neurons. Myelin also provides a conduit for energy transfer by oligodendrocytes to neurons. In the CNS, oligodendrocyte and axon are closely connected, which facilitating the crosstalk between neuron and oligodendrocyte (55). Both neuronal and oligodendroglial homeostasis are related. Axonal injury leads to demyelination while demyelination could trigger neurodegeneration (56).

**Figure 1:**
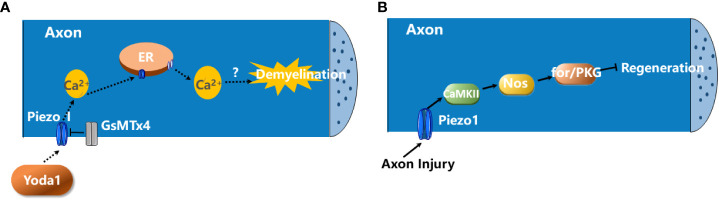
The roles of Piezo1 in demyelination and axon degeneration. **(A)** *The activation of Piezo1 channels in axon negatively regulates CNS myelination.* The activation of Piezo1 channels in axon by Yoda1 promotes the influx of extracellular Ca^2+^ into the neuron which, in turn, triggers Ca^2+^-induced Ca^2+^ release (CICR) from ER. This contributes to the demyelination of CNS axons. GsMTx4 blocks Piezo1 activity and prevents the demyelination (57). **(B)** *Piezo1 activation inhibits axon regeneration.* Upon axon injury, Piezo1 is recruited to the growth cones and inhibits axon regeneration *via* the CaMKII-Nos-PKG pathway (58).

It has showed that activation of Piezo1 channels in axon negatively regulates CNS myelination (57). Piezo1 is highly expressed in neurons but not in mature oligodendrocytes. The application of Yoda-1, which is a positive modulator of Piezo1 channel opening, induces demyelination (**Figure 1A**). In contrast, the Piezo1 antagonist GsMTx4 attenuates demyelination induced by psychosine, a cytotoxic lipid, in an *ex vivo* murine-derived organotypic cerebellar slice culture model and lysophosphatidylcholine (LPC)-induced demyelination animal model (57).

In addition, Piezo1 activation inhibits axon regeneration. Upon axon injury, Piezo1 is recruited to the growth cone and activated, which induces Ca^2+^ transient and Ca^2+^/calmodulin-dependent protein kinase II (CaMKII) activation, leading to activation of nitric oxide synthase (NOS) and the downstream cGMP kinase Foraging or PKG to restrict axon regrowth (**Figure 1B**) (58). But whether this signaling pathway occurs in MS remains unknown.

### Microglia and macrophages

Macrophages and microglia are innate immune cells that perform diverse functions including homeostasis, pathogen defense, and response to injury. These cells play important roles in inflammation and/or wound healing. They have the ability to produce reactive oxygen species (ROS), reactive nitrogen species (RNS), and pro-inflammatory cytokines and chemokines during the progression of MS. In EAE, NADPH oxidase (NOX) activity remains high in microglia even after the peak of the disease (59). In addition, these cells adopt multiple states, including pro-inflammatory and anti-inflammatory phenotypes. Among them, the anti-inflammatory phenotype favors oligodendrocyte differentiation and enhances remyelination in LPC induced demyelination mouse model (60). While the effects of biochemical cues, including cytokines and chemokines, on macrophage/microglial functions have been widely studied, the roles of physical cues, including mechanical stimuli, in regulating their function have only started to emerge. For example, macrophages cultured on soft substrates have reduced inflammation compared with cells adhered to stiff substrates (61).

**Figure 2:**
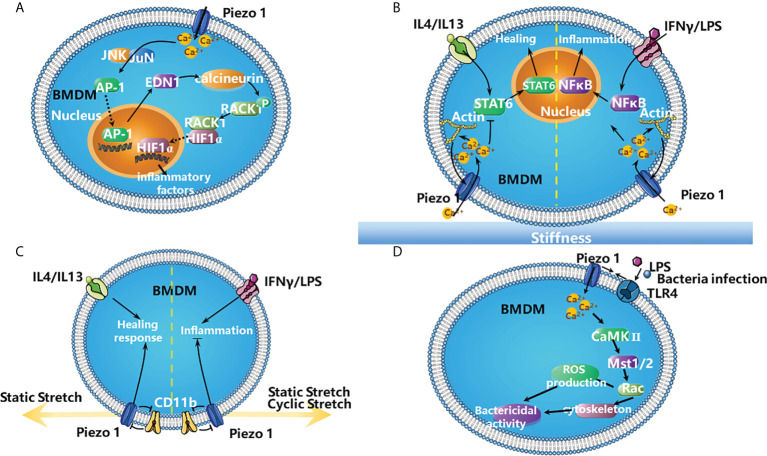
The roles of Piezo1 in the inflammation. **(A)** *The activation of Piezo1 in BMDMs by mechanical force favors inflammation.* Mechanical stimulation of BMDM triggers the expression of proinflammatory factors *via* Piezo1. Piezo1 activation increases the concentration of Ca^2+^ in the cytosol, which increases the activity of AP-1 and Edn1 production. Edn1 signalling in turn stabilizes HIF1α by activating calcineurin, which dephosphorylates RACK1. The stabilization of HIF1α favors the productions of the proinflammatory factors (62). **(B)** *Stiffness-dependent modulation of Piezo1 activity regulates polarization responses of BMDMs.* Activation of Piezo1 promotes actin polymerization, which enhances channel mediated Ca^2+^ influx. It promotes IFNγ/LPS-induced inflammation *via* NFκB and suppresses IL4/IL13-induced healing responses by STAT6 in BMDMs (63). **(C)** *Piezo1 works with integrins to modulate macrophage inflammatory and healing responses.* Both static and cyclic stretch suppresses IFNγ/LPS induced inflammation. In contrast, IL4/IL13 mediated healing responses are enhanced with static stretch conditions. Knock down of either CD11b or Piezo1 abrogates these stretch-mediated changes in inflammatory responses. In addition, there is crosstalk between CD11b and Piezo1, knock down of CD11b enhances the expression of Piezo1, and conversely knock down of Piezo1 enhances CD11b expression (64). **(D)** *TLR4 signalling augments macrophage bactericidal activity through Piezo1.* Bacterial infection or LPS induces the assembly of Piezo1 and TLR4, these changes increase the ability of macrophages to destroy bacterial *via* producing more ROS and remodelling cytoskeleton. It is possible that LPS stimulates TLR4 to induce Piezo1-mediated Ca^2+^ influx and consequently activates CaMKII- Mst1/2-Rac axis to remove pathogen (65).

Several recent studies have investigated the roles of Piezo1 in the functions of macrophages. Piezo1 activation in macrophages by mechanical force promotes inflammation. Upon exposure to cyclical hydrostatic pressure (CHP), bone-marrow derived macrophages (BMDMs) upregulated the expression of pro-inflammatory genes, most of which were targets of hypoxia-inducible factor-1-alpha (HIF1α), which requires the activation of Piezo1. The authors proposed that the activation of Piezo1 by CHP enhances the activity of activating protein-1 (AP-1) and transcription of endothelin-1 (*Edn1*), which is Ca^2+^ dependent. EDN1 signalling in turn stabilizes HIF1α, favoring the proinflammatory expression profile in BMDMs (**Figure 2A**) (62).

Piezo1 is also involved in macrophage polarization and detection of microenvironmental stiffness. In BMDMs, Piezo1 enhances IFNγ- and lipopolysaccharide (LPS)-induced inflammation and reduces interleukin-4 (IL-4)- and IL-13-induced healing responses (**Figure 2B**). In addition, Piezo1 also modulates macrophage functions depending on microenvironmental stiffness *in vitro* and affects the immune responses to subcutaneous implantation of biomaterials *in vivo*. There are positive feedback regulations between Piezo1 and actin that favor macrophage inflammatory activation. Piezo1 enhances actin polymerization, and the actin cytoskeleton promotes Piezo1-mediated Ca^2+^ activity (**Figure 2B**) (63).

Furthermore, Piezo1 together with integrins modulate macrophage inflammatory and healing responses. Stretch as well as biochemical stimuli alter the expression of inflammatory or healing responses in macrophages. During stretch, CD11b (a highly expressed integrin in macrophages) expression is enhanced while Piezo1 expression is reduced. Knocking down either CD11b or Piezo1 through small interfering RNA (siRNA) abrogates stretch-mediated changes in inflammatory responses. Moreover, CD11b knockdown enhances the expression of Piezo1 and inflammation, while Piezo1 knockdown enhances CD11b expression and suppress inflammation (**Figure 2C**) (64).

Furthermore, Toll like receptor 4 (TLR4) signalling augments macrophage bactericidal activity through Piezo1. Bacterial infection or LPS induces the assembly of Piezo1 and TLR4 and remodels F-actin; these changes increase the ability of macrophages to destroy bacterial and to produce more ROS. Genetic deficiency of Piezo1 abrogates these responses. It is possible that LPS stimulates TLR4 to induce Piezo1-mediated Ca^2+^ influx and consequently activates CaMKII–macrophage stimulating 1/2 (Mst1/2)–Rac axis to remove pathogens (**Figure 2D**) (65).

**Figure 3:**
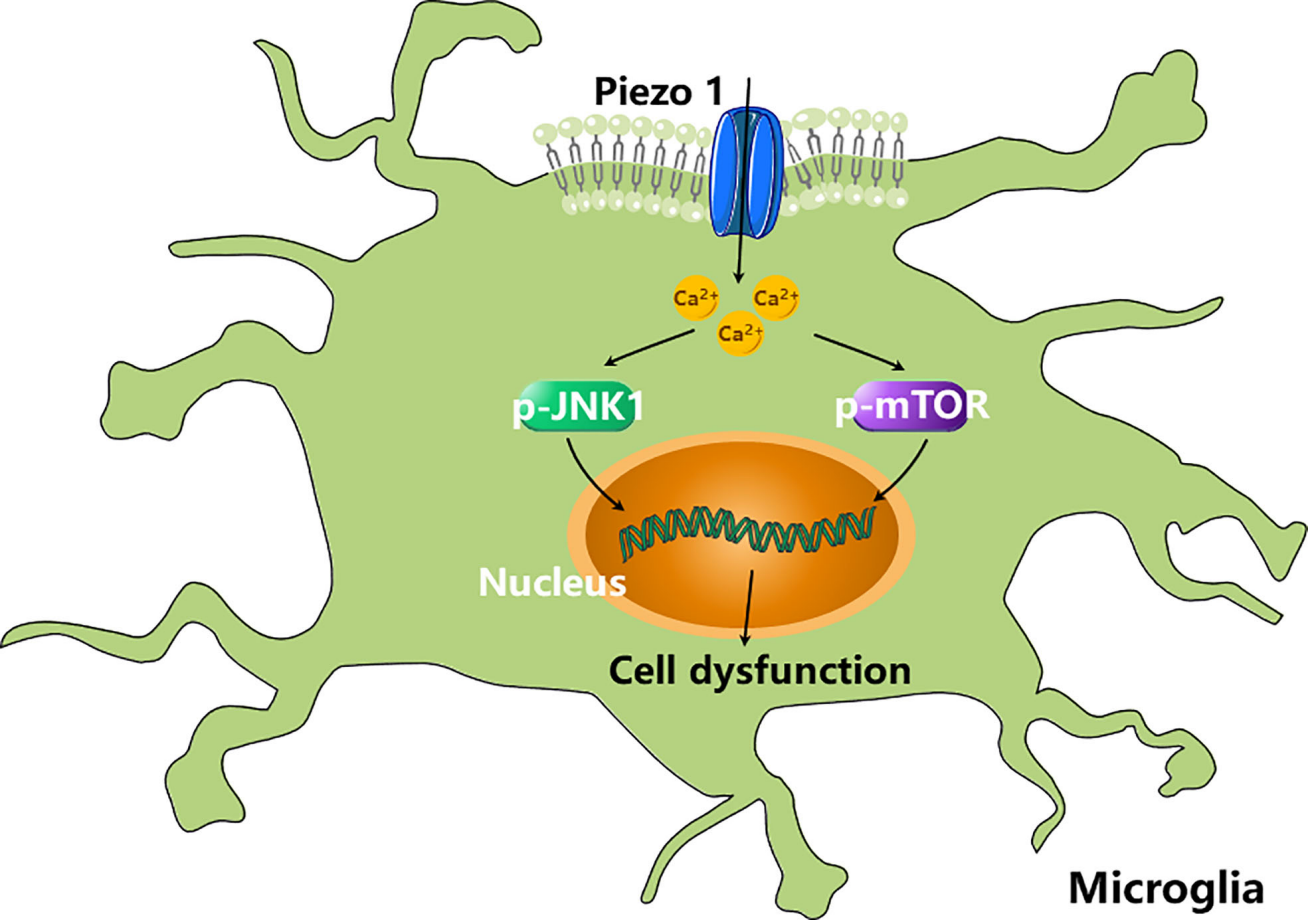
Piezo1 activation modulates microglial activity. High concentration of glucose (HCG) increases the expression of Piezo1 in microglia, which increases cytosolic Ca^2+^ signalling and reduces the phosphorylation of mTOR and JNK1, triggering cell dysfunction (66).

The role of Piezo1 in microglia has also been studied. Piezo1 activation modulates microglial activity in an acute hyperglycemia model. A high glucose concentration increases the expression of Piezo1 in microglia, which increases cytosolic Ca^2+^ signaling and reduces the phosphorylation of mTOR and JNK1, triggering cell dysfunction (**Figure 3**) (66). Although these studies have shown that Piezo1 channels in macrophages/microglia are involved in inflammation, the exact roles of Piezo1 in EAE inflammation remains unknown.

### T cells

In both MS and EAE, the immunopathological processes are initiated by the activation of T helper type 1 (Th1) and T helper type 17 (Th17) cells in the periphery. Upon crossing the BBB, these T cells are restimulated by APCs in the brain. They then migrate into the parenchyma and produce proinflammatory cytokines— IFN𝛾 and IL-17—to destroy myelin.

**Figure 4:**
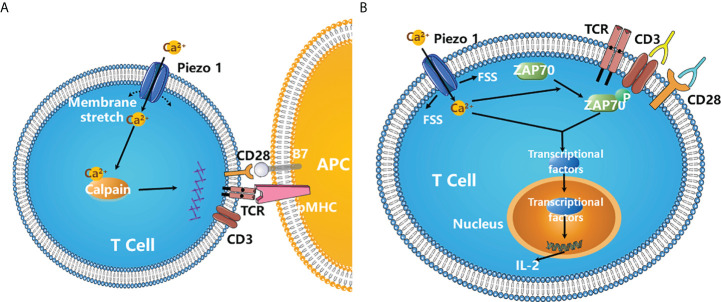
Piezo1 is required for the optimal T cell activation. **(A)** During immune synapse formation, membrane stretch triggers Piezo1 opening and Ca^2+^ influx, which results in the activation of calpain. Activated calpain then reorganizes cortical actin scaffold, thereby favoring the optimal human T cell activation (68). **(B)** Fluid shear stress (FSS) in combination with soluble and/or bead-bound CD3/CD28 Abs significantly enhanced the activation of T cells and ZAP70 phosphorylation, which requires the activation of Piezo1 channels and Ca^2+^ influx. FSS also activates three major transcription factor NFAT, NF-κB and AP-1, increasing the expression of cytokines such as IL-2 (69). .

Usually, the interaction between T cell receptors (TCRs) and peptides on MHC molecules triggers T cell activation. The strength of the interaction determines the extent of T cell activation. Recent studies have shown that mechanical forces also contribute to optimal T cell activation. For example, antibodies (Abs) immobilized on beads induce TCR activation more efficiently than soluble Abs (67). Not surprisingly, Piezo1 is involved in human T cell activation. Immobilized cross-linked Abs cannot induce maximal TCR activation in CD4+ and CD8+ T cells without Piezo1, but chemical activation of Piezo1 by Yoda1 can rescue this dysfunction. In addition, the inhibition of calpain prevents optimal TCR activation. In T cells, Ca^2+^ influx is quickly induced by TCR cross-linking by bead-bound CD3/CD28 Abs, which depends on Piezo1. It is proposed that mechanical stretch during immune synapse formation triggers Piezo1 activation and Ca^2+^ influx, inducing calpain activation, which contributes to optimal TCR signaling (**Figure 4A**) (68). Additionally, in Jurkat and primary human T cells, fluid shear stress (FSS) in combination with soluble and/or bead-bound CD3/CD28 Abs enhances the activation of T cells, which relies on Ca^2+^ and Piezo1 activation (**Figure 4B**) (69).

The role of Piezo1 in CD4+ T cells in EAE mice has been studied. Piezo1 deletion in CD4+ T cells reduces disease severity in EAE mice. However, it does not affect thymic development, lymph node homing, TCR priming, T cell proliferation and differentiation. These results are different from the conclusions reported by Liu et al, 2018 (68), which might be caused by the specific methods they used (siRNA versus genetic knockout) or T cells from different species (human versus mouse). In addition, Piezo1 deletion in CD4+ T cells expands the pool of Tregs, indicating Piezo1 inhibits Treg activity. Moreover, when Piezo1 is specifically deleted in Tregs cells, it significantly attenuates EAE symptoms (70). It is possible that when T cells navigate through stiff environments in MS/EAE, membrane tension of T cells activates Piezo1 channels. After Piezo1 are activated, they inhibit TGFβ/SMAD signaling pathway in CD4+ T cells and restrain Treg activity (70), which impairs the ability of Treg cells to suppress autoreactive effector T cells in MS/EAE (71).

## 5. Conclusion

Current research indicates that Piezo1 channels have key roles in multiple aspects of MS pathogenesis. Activation of Piezo1 channels in axon negatively regulates CNS myelination. In addition, Piezo1 inhibition in CD4+ T cells and/or Tregs attenuates EAE symptoms. However, the effects of Piezo1 on inflammation are complex: While Piezo1 activation is protective against bacteria infection, deletion of Piezo1 is also protective in a mouse model of pulmonary fibrosis (62). Furthermore, although MS is an inflammatory disease, the role of Piezo1 in MS inflammation has yet to be clarified. The analog of Piezo1, Piezo2, is also expressed in the nervous system. It showed that both Piezo1 and Piezo2 are highly expressed in Schwann cells, but they have different roles in myelination. Piezo1 inhibits radial and longitudinal myelination while Piezo2 is required for myelin formation in the peripheral nervous system (PNS) (72). But whether Piezo2 is involved in MS remains unstudied.

In addition, both activators and blockers of Piezo1 have low affinity for Piezo1; these drugs have poor solubility and stability, so their usage *in vivo* has been limited. Hence, more work is required to identify additional Piezo1 modulators.

## Author contributions

KY and XH reviewed the literature, and drafted and wrote the manuscript. ZW, YY, HP, and XZ revised the manuscript. TS proposed the topic of this manuscript and revised the manuscript. All authors read and approved the final version.

## Funding

This work was supported by the National Natural Science Foundation of China to TS (51873168).

## Conflict of interest

The authors declare that the research was conducted in the absence of any commercial or financial relationships that could be construed as a potential conflict of interest.

## Publisher’s note

All claims expressed in this article are solely those of the authors and do not necessarily represent those of their affiliated organizations, or those of the publisher, the editors and the reviewers. Any product that may be evaluated in this article, or claim that may be made by its manufacturer, is not guaranteed or endorsed by the publisher.

## References

[1] Filippi M Bar-Or A Piehl F Preziosa P Solari A Vukusic S. Multiple sclerosis. Nat Rev Dis Primers (2018) 4(1):43. doi: 10.1038/s41572-018-0041-4 30410033

[2] Yong HYF Yong VW. Mechanism-based criteria to improve therapeutic outcomes in progressive multiple sclerosis. Nat Rev Neurol (2022) 18(1):40–55. doi: 10.1038/s41582-021-00581-x 34732831

[3] Munger KL Hongell K Aivo J Soilu-Hanninen M Surcel HM Ascherio A. 25-hydroxyvitamin d deficiency and risk of MS among women in the Finnish maternity cohort. Neurology (2017) 89(15):1578–83. doi: 10.1212/WNL.0000000000004489 PMC5634665 28904091

[4] Munger KL Hongell K Cortese M Aivo J Soilu-Hanninen M Surcel HM. Epstein-Barr virus and multiple sclerosis risk in the finnish maternity cohort. Ann Neurol (2019) 86(3):436–42. doi: 10.1002/ana.25532 PMC6839107 31226219

[5] Szymczak I Pawliczak R. The active metabolite of vitamin D3 as a potential immunomodulator. Scand J Immunol (2016) 83(2):83–91. doi: 10.1111/sji.12403 26678915

[6] Cotsapas C Mitrovic M. Genome-wide association studies of multiple sclerosis. Clin Transl Immunol (2018) 7(6):e1018. doi: 10.1002/cti2.1018 PMC5983059 29881546

[7] Titus HE Chen Y Podojil JR Robinson AP Balabanov R Popko B. Pre-clinical and clinical implications of "Inside-out" vs. "Outside-in" paradigms in multiple sclerosis etiopathogenesis. Front Cell Neurosci (2020) 14:599717. doi: 10.3389/fncel.2020.599717 33192332 PMC7654287

[8] t Hart BA Luchicchi A Schenk GJ Stys PK Geurts JJG. Mechanistic underpinning of an inside-out concept for autoimmunity in multiple sclerosis. Ann Clin Transl Neurol (2021) 8(8):1709–19. doi: 10.1002/acn3.51401 PMC8351380 34156169

[9] Sen MK Almuslehi MSM Shortland PJ Coorssen JR Mahns DA. Revisiting the pathoetiology of multiple sclerosis: Has the tail been wagging the mouse? Front Immunol (2020) 11:572186. doi: 10.3389/fimmu.2020.572186 33117365 PMC7553052

[10] Constantinescu CS Farooqi N O'Brien K Gran B. Experimental autoimmune encephalomyelitis (EAE) as a model for multiple sclerosis (MS). Br J Pharmacol (2011) 164(4):1079–106. doi: 10.1111/j.1476-5381.2011.01302.x PMC3229753 21371012

[11] Havrdova E Horakova D Kovarova I. Alemtuzumab in the treatment of multiple sclerosis: key clinical trial results and considerations for use. Ther Adv Neurol Disord (2015) 8(1):31–45. doi: 10.1177/1756285614563522 25584072 PMC4286943

[12] Khoy K Mariotte D Defer G Petit G Toutirais O Le Mauff B. Natalizumab in multiple sclerosis treatment: From biological effects to immune monitoring. Front Immunol (2020) 11:549842. doi: 10.3389/fimmu.2020.549842 33072089 PMC7541830

[13] Lucchinetti CF Popescu BF Bunyan RF Moll NM Roemer SF Lassmann H. Inflammatory cortical demyelination in early multiple sclerosis. N Engl J Med (2011) 365(23):2188–97. doi: 10.1056/NEJMoa1100648 PMC3282172 22150037

[14] Pardini M Brown JWL Magliozzi R Reynolds R Chard DT. Surface-in pathology in multiple sclerosis: a new view on pathogenesis? Brain (2021) 144(6):1646–54. doi: 10.1093/brain/awab025 33876200

[15] Keating CE Cullen DK. Mechanosensation in traumatic brain injury. Neurobiol Dis (2021) 148:105210. doi: 10.1016/j.nbd.2020.105210 33259894 PMC7847277

[16] Murphy MC Huston J 3rd Ehman RL. MR elastography of the brain and its application in neurological diseases. Neuroimage (2019) 187:176–83. doi: 10.1016/j.neuroimage.2017.10.008 PMC5889749 28993232

[17] Viji Babu PK Radmacher M. Mechanics of brain tissues studied by atomic force microscopy: A perspective. Front Neurosci (2019) 13:600. doi: 10.3389/fnins.2019.00600 31258462 PMC6587663

[18] Christ AF Franze K Gautier H Moshayedi P Fawcett J Franklin RJ. Mechanical difference between white and gray matter in the rat cerebellum measured by scanning force microscopy. J Biomech. (2010) 43(15):2986–92. doi: 10.1016/j.jbiomech.2010.07.002 20656292

[19] van Dommelen JA van der Sande TP Hrapko M Peters GW. Mechanical properties of brain tissue by indentation: interregional variation. J Mech Behav BioMed Mater. (2010) 3(2):158–66. doi: 10.1016/j.jmbbm.2009.09.001 20129415

[20] Kaster T Sack I Samani A. Measurement of the hyperelastic properties of *ex vivo* brain tissue slices. J Biomech. (2011) 44(6):1158–63. doi: 10.1016/j.jbiomech.2011.01.019 21329927

[21] Finan JD Elkin BS Pearson EM Kalbian IL Morrison B 3rd. Viscoelastic properties of the rat brain in the sagittal plane: effects of anatomical structure and age. Ann BioMed Eng (2012) 40(1):70–8. doi: 10.1007/s10439-011-0394-2 22012082

[22] Elkin BS Morrison B. Viscoelastic properties of the P17 and adult rat brain from indentation in the coronal plane. J Biomech. Eng (2013) 135(11):114507. doi: 10.1115/1.4025386 24026193

[23] Budday S Nay R de Rooij R Steinmann P Wyrobek T Ovaert TC. Mechanical properties of gray and white matter brain tissue by indentation. J Mech Behav BioMed Mater. (2015) 46:318–30. doi: 10.1016/j.jmbbm.2015.02.024 PMC4395547 25819199

[24] Weickenmeier J de Rooij R Budday S Steinmann P Ovaert TC Kuhl E. Brain stiffness increases with myelin content. Acta Biomater. (2016) 42:265–72. doi: 10.1016/j.actbio.2016.07.040 27475531

[25] Forte AE Gentleman SM Dini D. On the characterization of the heterogeneous mechanical response of human brain tissue. Biomech. Model Mechanobiol. (2017) 16(3):907–20. doi: 10.1007/s10237-016-0860-8 PMC5422507 27933417

[26] Samadi-Dooki A Voyiadjis GZ Stout RW. An indirect indentation method for evaluating the linear viscoelastic properties of the brain tissue. J Biomech. Eng (2017) 139(6). doi: 10.1115/1.4036486 28418454

[27] Luque T Kang MS Schaffer DV Kumar S. Microelastic mapping of the rat dentate gyrus. R Soc Open Sci (2016) 3(4):150702. doi: 10.1098/rsos.150702 27152213 PMC4852636

[28] Koser DE Moeendarbary E Hanne J Kuerten S Franze K. CNS cell distribution and axon orientation determine local spinal cord mechanical properties. Biophys J (2015) 108(9):2137–47. doi: 10.1016/j.bpj.2015.03.039 PMC4423070 25954872

[29] Green MA Bilston LE Sinkus R. *In vivo* brain viscoelastic properties measured by magnetic resonance elastography. NMR BioMed (2008) 21(7):755–64. doi: 10.1002/nbm.1254 18457350

[30] Feng Y Clayton EH Chang Y Okamoto RJ Bayly PV. Viscoelastic properties of the ferret brain measured *in vivo* at multiple frequencies by magnetic resonance elastography. J Biomech. (2013) 46(5):863–70. doi: 10.1016/j.jbiomech.2012.12.024 PMC3616770 23352648

[31] Sack I Streitberger KJ Krefting D Paul F Braun J. The influence of physiological aging and atrophy on brain viscoelastic properties in humans. PLoS One (2011) 6(9):e23451. doi: 10.1371/journal.pone.0023451 21931599 PMC3171401

[32] Lipp A Trbojevic R Paul F Fehlner A Hirsch S Scheel M. Cerebral magnetic resonance elastography in supranuclear palsy and idiopathic parkinson's disease. NeuroImage Clin (2013) 3:381–7. doi: 10.1016/j.nicl.2013.09.006 PMC3814959 24273721

[33] Murphy MC Huston J 3rd Jack CR Jr. Glaser KJ Manduca A Felmlee JP. Decreased brain stiffness in alzheimer's disease determined by magnetic resonance elastography. J Magn Reson Imaging (2011) 34(3):494–8. doi: 10.1002/jmri.22707 PMC3217096 21751286

[34] Wuerfel J Paul F Beierbach B Hamhaber U Klatt D Papazoglou S. MR-elastography reveals degradation of tissue integrity in multiple sclerosis. Neuroimage (2010) 49(3):2520–5. doi: 10.1016/j.neuroimage.2009.06.018 19539039

[35] Streitberger KJ Sack I Krefting D Pfuller C Braun J Paul F. Brain viscoelasticity alteration in chronic-progressive multiple sclerosis. PLoS One (2012) 7(1):e29888. doi: 10.1371/journal.pone.0029888 22276134 PMC3262797

[36] Fehlner A Behrens JR Streitberger KJ Papazoglou S Braun J Bellmann-Strobl J. Higher-resolution MR elastography reveals early mechanical signatures of neuroinflammation in patients with clinically isolated syndrome. J Magn Reson Imaging (2016) 44(1):51–8. doi: 10.1002/jmri.25129 26714969

[37] Riek K Millward JM Hamann I Mueller S Pfueller CF Paul F. Magnetic resonance elastography reveals altered brain viscoelasticity in experimental autoimmune encephalomyelitis. NeuroImage Clin (2012) 1(1):81–90. doi: 10.1016/j.nicl.2012.09.003 24179740 PMC3757734

[38] Silva RV Morr AS Mueller S Koch SP Boehm-Sturm P Rodriguez-Sillke Y. Contribution of tissue inflammation and blood-brain barrier disruption to brain softening in a mouse model of multiple sclerosis. Front Neurosci (2021) 15:701308. doi: 10.3389/fnins.2021.701308 34497486 PMC8419310

[39] Millward JM Guo J Berndt D Braun J Sack I Infante-Duarte C. Tissue structure and inflammatory processes shape viscoelastic properties of the mouse brain. NMR BioMed (2015) 28(7):831–9. doi: 10.1002/nbm.3319 25963743

[40] Pyka-Fosciak G Zemla J Lis GJ Litwin JA Lekka M. Changes in spinal cord stiffness in the course of experimental autoimmune encephalomyelitis, a mouse model of multiple sclerosis. Arch Biochem Biophys (2020) 680:108221. doi: 10.1016/j.abb.2019.108221 31816310

[41] Schregel K Wuerfel E Garteiser P Gemeinhardt I Prozorovski T Aktas O. Demyelination reduces brain parenchymal stiffness quantified *in vivo* by magnetic resonance elastography. Proc Natl Acad Sci U.S.A. (2012) 109(17):6650–5. doi: 10.1073/pnas.1200151109 PMC3340071 22492966

[42] Urbanski MM Brendel MB Melendez-Vasquez CV. Acute and chronic demyelinated CNS lesions exhibit opposite elastic properties. Sci Rep (2019) 9(1):999. doi: 10.1038/s41598-018-37745-7 30700777 PMC6354022

[43] Coste B Mathur J Schmidt M Earley TJ Ranade S Petrus MJ. Piezo1 and Piezo2 are essential components of distinct mechanically activated cation channels. Science (2010) 330(6000):55–60. doi: 10.1126/science.1193270 20813920 PMC3062430

[44] Saotome K Murthy SE Kefauver JM Whitwam T Patapoutian A Ward AB. Structure of the mechanically activated ion channel Piezo1. Nature (2018) 554(7693):481–6. doi: 10.1038/nature25453 PMC6010196 29261642

[45] Zhao Q Zhou H Chi S Wang Y Wang J Geng J. Structure and mechanogating mechanism of the Piezo1 channel. Nature (2018) 554(7693):487–92. doi: 10.1038/nature25743 29469092

[46] Guo YR MacKinnon R. Structure-based membrane dome mechanism for piezo mechanosensitivity. Elife (2017) 6. doi: 10.7554/eLife.33660 PMC5788504 29231809

[47] Haselwandter CA MacKinnon R. Piezo's membrane footprint and its contribution to mechanosensitivity. Elife (2018) 7. doi: 10.7554/eLife.41968 PMC6317911 30480546

[48] Ranade SS Syeda R Patapoutian A. Mechanically Activated Ion Channels. Neuron. (2015) 87(6):1162–79. doi: 10.1016/j.neuron.2015.08.032 PMC4582600 26402601

[49] Cox CD Bae C Ziegler L Hartley S Nikolova-Krstevski V Rohde PR. Removal of the mechanoprotective influence of the cytoskeleton reveals PIEZO1 is gated by bilayer tension. Nat Commun (2016) 7:10366. doi: 10.1038/ncomms10366 26785635 PMC4735864

[50] Syeda R Florendo MN Cox CD Kefauver JM Santos JS Martinac B. Piezo1 channels are inherently mechanosensitive. Cell Rep (2016) 17(7):1739–46. doi: 10.1016/j.celrep.2016.10.033 PMC5129625 27829145

[51] Gottlieb PA Bae C Sachs F. Gating the mechanical channel Piezo1: a comparison between whole-cell and patch recording. Channels (Austin) (2012) 6(4):282–9. doi: 10.4161/chan.21064 PMC3508907 22790451

[52] Wetzel C Hu J Riethmacher D Benckendorff A Harder L Eilers A. A stomatin-domain protein essential for touch sensation in the mouse. Nature (2007) 445(7124):206–9. doi: 10.1038/nature05394 17167420

[53] Qi Y Andolfi L Frattini F Mayer F Lazzarino M Hu J. Membrane stiffening by STOML3 facilitates mechanosensation in sensory neurons. Nat Commun (2015) 6:8512. doi: 10.1038/ncomms9512 26443885 PMC4633829

[54] Nourse JL Pathak MM. How cells channel their stress: Interplay between Piezo1 and the cytoskeleton. Semin Cell Dev Biol (2017) 71:3–12. doi: 10.1016/j.semcdb.2017.06.018 28676421 PMC6070642

[55] Chang KJ Redmond SA Chan JR. Remodeling myelination: implications for mechanisms of neural plasticity. Nat Neurosci (2016) 19(2):190–7. doi: 10.1038/nn.4200 PMC4792270 26814588

[56] Simons M Misgeld T Kerschensteiner M. A unified cell biological perspective on axon-myelin injury. J Cell Biol (2014) 206(3):335–45. doi: 10.1083/jcb.201404154 PMC4121977 25092654

[57] Velasco-Estevez M Gadalla KKE Linan-Barba N Cobb S Dev KK Sheridan GK. Inhibition of Piezo1 attenuates demyelination in the central nervous system. Glia (2020) 68(2):356–75. doi: 10.1002/glia.23722 31596529

[58] Song Y Li D Farrelly O Miles L Li F Kim SE. The mechanosensitive ion channel piezo inhibits axon regeneration. Neuron (2019) 102(2):373–89.e376. doi: 10.1016/j.neuron.2019.01.050 30819546 PMC6487666

[59] Di Filippo M de Iure A Giampa C Chiasserini D Tozzi A Orvietani PL. Persistent activation of microglia and NADPH oxidase [corrected] drive hippocampal dysfunction in experimental multiple sclerosis. Sci Rep (2016) 6:20926. doi: 10.1038/srep20926 26887636 PMC4757867

[60] Miron VE Boyd A Zhao JW Yuen TJ Ruckh JM Shadrach JL. M2 microglia and macrophages drive oligodendrocyte differentiation during CNS remyelination. Nat Neurosci (2013) 16(9):1211–8. doi: 10.1038/nn.3469 PMC3977045 23872599

[61] Blakney AK Swartzlander MD Bryant SJ. The effects of substrate stiffness on the *in vitro* activation of macrophages and *in vivo* host response to poly(ethylene glycol)-based hydrogels. J BioMed Mater. Res A (2012) 100(6):1375–86. doi: 10.1002/jbm.a.34104 PMC3339197 22407522

[62] Solis AG Bielecki P Steach HR Sharma L Harman CCD Yun S. Mechanosensation of cyclical force by PIEZO1 is essential for innate immunity. Nature (2019) 573(7772):69–74. doi: 10.1038/s41586-019-1485-8 31435009 PMC6939392

[63] Atcha H Jairaman A Holt JR Meli VS Nagalla RR Veerasubramanian PK. Mechanically activated ion channel Piezo1 modulates macrophage polarization and stiffness sensing. Nat Commun (2021) 12(1):3256. doi: 10.1038/s41467-021-23482-5 34059671 PMC8167181

[64] Atcha H Meli VS Davis CT Brumm KT Anis S Chin J. Crosstalk between CD11b and Piezo1 mediates macrophage responses to mechanical cues. Front Immunol (2021) 12:689397. doi: 10.3389/fimmu.2021.689397 34630381 PMC8493066

[65] Geng J Shi Y Zhang J Yang B Wang P Yuan W. TLR4 signalling *via* Piezo1 engages and enhances the macrophage mediated host response during bacterial infection. Nat Commun (2021) 12(1):3519. doi: 10.1038/s41467-021-23683-y 34112781 PMC8192512

[66] Liu H Bian W Yang D Yang M Luo H. Inhibiting the Piezo1 channel protects microglia from acute hyperglycaemia damage through the JNK1 and mTOR signalling pathways. Life Sci (2021) 264:118667. doi: 10.1016/j.lfs.2020.118667 33127514

[67] Geppert TD Lipsky PE. Activation of T lymphocytes by immobilized monoclonal antibodies to CD3. regulatory influences of monoclonal antibodies to additional T cell surface determinants. J Clin Invest (1988) 81(5):1497–505. doi: 10.1172/JCI113481 PMC442582 2452835

[68] Liu CSC Raychaudhuri D Paul B Chakrabarty Y Ghosh AR Rahaman O. Cutting edge: Piezo1 mechanosensors optimize human T cell activation. J Immunol (2018) 200(4):1255–60. doi: 10.4049/jimmunol.1701118 29330322

[69] Hope JM Dombroski JA Pereles RS Lopez-Cavestany M Greenlee JD Schwager SC. Fluid shear stress enhances T cell activation through Piezo1. BMC Biol (2022) 20(1):61. doi: 10.1186/s12915-022-01266-7 35260156 PMC8904069

[70] Jairaman A Othy S Dynes JL Yeromin AV Zavala A Greenberg ML. Piezo1 channels restrain regulatory T cells but are dispensable for effector CD4(+) T cell responses. Sci Adv (2021) 7(28). doi: 10.1126/sciadv.abg5859 PMC8262815 34233878

[71] Othy S Jairaman A Dynes JL Dong TX Tune C Yeromin AV. Regulatory T cells suppress Th17 cell Ca(2+) signaling in the spinal cord during murine autoimmune neuroinflammation. Proc Natl Acad Sci U.S.A. (2020) 117(33):20088–99. doi: 10.1073/pnas.2006895117 PMC7443932 32732436

[72] Acheta J Bhatia U Haley J Hong J Rich K Close R. Piezo channels contribute to the regulation of myelination in schwann cells. Glia (2022). doi: 10.1002/glia.24251 PMC10638658 35903933

